# Luman is involved in osteoclastogenesis through the regulation of DC-STAMP expression, stability and localization

**DOI:** 10.1242/jcs.176057

**Published:** 2015-12-01

**Authors:** Soshi Kanemoto, Yasuhiro Kobayashi, Teruhito Yamashita, Takeshi Miyamoto, Min Cui, Rie Asada, Xiang Cui, Kenta Hino, Masayuki Kaneko, Tomoko Takai, Koji Matsuhisa, Naoyuki Takahashi, Kazunori Imaizumi

**Affiliations:** 1 Department of Biochemistry, Institute of Biomedical and Health Sciences, Hiroshima University, 1-2-3 Kasumi, Minami-ku, Hiroshima 734-8553, Japan; 2 Institute for Oral Science, Matsumoto Dental University, 1780 Gobara, Hiro-oka, Shiojiri, Nagano 399-0781, Japan; 3 Department of Orthopedic Surgery, Keio University School of Medicine, 35 Shinanomachi, Shinjuku-ku, Tokyo 160-8582, Japan

**Keywords:** Osteoclastogenesis, Cell-cell fusion, Endoplasmic reticulum, Transcription factor

## Abstract

Luman (also known as CREB3) is a type-II transmembrane transcription factor belonging to the OASIS family that localizes to the endoplasmic reticulum (ER) membrane under normal conditions. In response to ER stress, OASIS-family members are subjected to regulated intramembrane proteolysis (RIP), following which the cleaved N-terminal fragments translocate to the nucleus. In this study, we show that treatment of bone marrow macrophages (BMMs) with cytokines – macrophage colony-stimulating factor (M-CSF) and RANKL (also known as TNFSF11) – causes a time-dependent increase in Luman expression, and that Luman undergoes RIP and becomes activated during osteoclast differentiation. Small hairpin (sh)RNA-mediated knockdown of Luman in BMMs prevented the formation of multinucleated osteoclasts, concomitant with the suppression of DC-STAMP, a protein that is essential for cell–cell fusion in osteoclastogenesis. The N-terminus of Luman facilitates promoter activity of DC-STAMP, resulting in upregulation of DC-STAMP expression. Furthermore, Luman interacts with DC-STAMP, and controls its stability and localization. These results suggest that Luman regulates the multinucleation of osteoclasts by promoting cell fusion of mononuclear osteoclasts through DC-STAMP induction and intracellular distribution during osteoclastogenesis.

## Abstract (precis)

**Highlighted Article:** Luman, an ER-resident transcription factor, regulates the multinucleation of osteoclasts by promoting cell fusion of mononuclear osteoclasts through DC-STAMP induction and transportation during osteoclastogenesis.

## INTRODUCTION

Osteoclasts are unique bone-resorbing cells that differentiate from monocyte–macrophage lineage cells in response to receptor activator of nuclear factor kappa-B ligand (RANKL; also known as TNFSF11), which is produced in osteoblasts, osteocytes and bone marrow stromal cells (Suda et al., 1999; Nakashima et al., 2012). RANKL, in turn, triggers the activation of cellular (c)-Fos and nuclear factor of activated T cells cytoplasmic 1 (NFATc1), both of which are required for osteoclastogenesis (Grigoriadis et al., 1994; Takayanagi et al., 2002). NFATc1 functions as a master regulator of osteoclast differentiation and is activated by RANKL-mediated dephosphorylation. Activated NFATc1 is transported to the nucleus where it induces the expression of genes involved in osteoclast differentiation, including tartrate-resistant acid phosphatase (TRAP; also known as ACP5), cathepsin K (CtsK), dendritic cell-specific transmembrane protein (DC-STAMP) and the d2 isoform of vacuolar ATPase V(0) domain (ATP6v0d2) (Hayman, 2008; Matsumoto et al., 2004; Yagi et al., 2007; Kim et al., 2008). TRAP-positive mononuclear precursors of osteoclasts fuse to form the mature multinuclear TRAP-positive osteoclast that is capable of bone resorption.

DC-STAMP is essential for cell–cell fusion of osteoclasts and foreign body giant cells (FBGCs) (Yagi et al., 2005). DC-STAMP is a putative seven-transmembrane protein that is expressed in dendritic cells (DCs) (Hartgers et al., 2000), and that localizes to both the plasma membrane and the endoplasmic reticulum (ER) membrane (Hartgers et al., 2000; Eleveld-Trancikova et al., 2005; Sawatani et al., 2008). Like osteoclasts, DCs are differentiated from the monocyte–macrophages lineage. In DC-STAMP-deficient mice, multinucleated osteoclasts are lost and bone-resorbing activity is significantly reduced, resulting in osteopetrosis (Yagi et al., 2005), indicating that DC-STAMP is essential for osteoclastogenesis and bone maintenance. Recently, it has been reported that DC-STAMP and osteoclast stimulatory transmembrane protein (OC-STAMP) cooperatively modulate cell–cell fusion for the multinucleation of osteoclasts (Miyamoto et al., 2012b). The expression of DC-STAMP and OC-STAMP are vigorously regulated by the RANKL–NFATc1 pathway (Yagi et al., 2007; Miyamoto et al., 2012b) and signal transducer and activator of transcription 6 (STAT6)–STAT1 signaling (Miyamoto et al., 2012a). Indeed, STAT1-deficient macrophages show enhanced cell–cell fusion and elevated DC-STAMP expression in FBGCs. In contrast, a lack of STAT6 causes an increase in STAT1 activation, significantly inhibiting cell–cell fusion and decreasing DC-STAMP and OC-STAMP expression in IL-4-induced FBGCs.

Luman, also known as CREB3 or LZIP, is a cyclic AMP response element (CRE)-binding protein belonging to the old astrocyte specifically induced substance (OASIS) family that has four other closely related members, including OASIS, BBF2H7, CREB-H and AIbZIP (Asada et al., 2011), and those proteins are involved in the differentiation of various cells. Previously, it has been reported that Luman undergoes regulated intramembrane proteolysis (RIP) in response to ER stress (Liang et al., 2006). During RIP, Luman is processed by site-1 protease (S1P; also known as MBTPS1) that is resident in the Golgi, and then N-terminal fragments of Luman harboring the basic leucine zipper (bZIP) domain are released from the Golgi membrane, and these N-terminal fragments translocate to the nucleus and function as transcription factors (Liang et al., 2006; Raggo et al., 2002). Luman was originally isolated as a protein that interacts with herpes simplex virus (HSV)-related host cell factor, a protein required by the HSV transactivator VP16 and involved in cell proliferation (Lu et al., 1997). Recently, it has been demonstrated that Luman interacts with DC-STAMP and that it is involved in DC differentiation (Eleveld-Trancikova et al., 2010). However, physiological functions of the interaction between Luman and DC-STAMP remain to be completely elucidated.

In the present study, we found that Luman is induced during osteoclastogenesis and is involved in cell–cell fusion through the induction of DC-STAMP expression. More importantly, Luman also regulates the localization and the stability of DC-STAMP by interacting with it. Our findings indicate that Luman plays a key role in osteoclast differentiation.

## RESULTS

### Luman is induced in osteoclastogenesis and processed in response to RANKL signaling

**Fig. 1.**
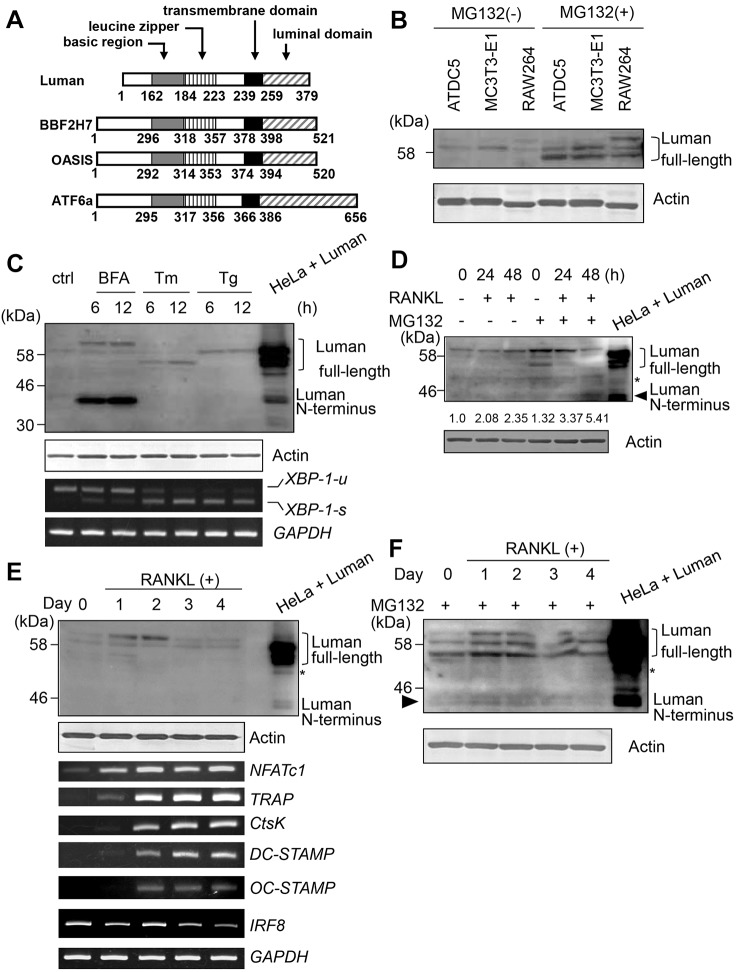
**Luman is induced during osteoclast differentiation and cleaved in response to RANKL signaling.** (A) Schematic representation of the domain structure of murine Luman, BBF2H7, OASIS and ATF6α. The basic leucine zipper (bZIP; basic region and leucine zipper), putative transmembrane domain and luminal domain are indicated. (B) Western blot analysis for Luman protein in cell lines originally derived from bone tissues: ATDC5, chondrocytes; MC3T3-E1, osteoblast-like cells; and RAW264, macrophages. Cells were cultured with or without 1 µM MG132 proteasome inhibitor for 6 h. Cell lysates were separated with SDS-PAGE, and western blotting was performed with an antibody against Luman. (C) RAW264 cells were cultured with 1 µM brefeldin A (BFA), 1 µg/ml tunicamycin (Tm), or 1 µM thapsigargin (Tg) for the indicated time periods. Western blotting was performed with an antibody against Luman (upper panel). Middle panel shows the expression levels of actin used as the internal control. Lower panels show RT-PCR analysis of *XBP-1* and *GAPDH* mRNA in each sample. Note that spliced forms of XBP-1 (*XBP-1-s*) were detected in cells that had been treated with tunicamycin and thapsigargin, but Luman N-termini were not detected under such conditions. (D) RAW264 cells were cultured with RANKL for the indicated time periods. Cell lysates were subjected to western blotting with an antibody against Luman. Relative quantified data of Luman N-terminus bands are indicated below the Luman blot. The intensity of the Luman N-terminus band in 0 h without MG132 treatment was set as 1.0. Arrowhead indicates Luman N-terminus bands. (E) BMMs were cultured with M-CSF and RANKL for the indicated time periods. Cell lysates were analyzed by western blotting with an antibody against Luman. The mRNA expression of the indicated genes was determined by using RT-PCR analysis. (F) BMMs were cultured with M-CSF and RANKL in the presence of 1 µM MG132 for 6 h. Cell lysates were analyzed by western blotting with an antibody against Luman. Note that the Luman N-terminus was detected in the presence of MG132 (arrowhead). For C–F, cell lysates from Luman-transfected HeLa cells were used as a positive control. Asterisk indicates non-specific bands.

Luman is a transmembrane transcription factor belonging to the OASIS family, the members of which are structurally very similar to ATF6, one of the ER stress transducers (Fig. 1A). We first checked the expression of Luman in cell lines derived from bone tissue – ATDC5, MC3T3-E1 and RAW264 cells. In all cell lines examined, the protein expression of Luman was weak under normal conditions. Because OASIS-family proteins are constitutively degraded by the ubiquitin-proteasome system (Kondo et al., 2012), we treated cells with the proteasome inhibitor MG132 (1 µM) and analyzed Luman expression using western blotting. Strong signals for the Luman protein were detected in all cell lines (Fig. 1B), indicating that Luman protein is rapidly degraded at a steady state, similar to other OASIS-family members. Double bands for full-length Luman were found in ATDC5 and MC3T3-E1 cells lysates, whereas triple bands were detected in the lysate of RAW264 cells, suggesting that Luman might be subject to different post-translational modifications, such as glycosylation in RAW264 cells, in contrast to ATDC5 or MC3T3-E1 cells. To examine whether Luman, like other OASIS-family members, is cleaved in response to ER stress, we treated RAW264 cells with two ER stressors – tunicamycin, which blocks N-linked glycosylation, and thapsigargin, which is a highly selective inhibitor of the ER Ca^2+^-dependent ATPase. Although Luman was cleaved upon treatment with brefeldin A (BFA), an agent that causes the reflux of Golgi enzymes – including S1P to the ER, as reported previously (Raggo et al., 2002) – we could not detect any cleavage of Luman upon treatment with ER stressors, even if proteasome inhibitor MG132 was used (Fig. 1C; Fig. S1A). This suggests that Luman can be processed by S1P at the Golgi, but that Luman is not cleaved in response to ER stress in RAW264 cells.

RAW264 macrophages can differentiate into osteoclasts upon stimulation with RANKL. We next analyzed the expression and cleavage of Luman in RAW264 cells after treatment with RANKL. Full-length Luman was upregulated at 24 and 48 h after RANKL stimulation (Fig. 1D). At the same time, cleaved fragments of Luman (the active form that is able to transcribe target genes) were also upregulated. We also stimulated bone marrow macrophages (BMMs) with RANKL and macrophage colony-stimulating factor (M-CSF) to initiate their differentiation into osteoclasts. *NFATc1* mRNA was upregulated and sustained at high levels after treatment with RANKL and M-CSF (Fig. 1E, lower panel). In addition, *TRAP* and *CtsK* were upregulated from day 2 of stimulation, as were *DC-STAMP* and *OC-STAMP*, which are involved in the cell–cell fusion step for multinucleated cell formation. On the contrary, mRNA of *IRF8*, which suppresses the differentiation of macrophages into osteoclasts (Zhao et al., 2009), decreased with the progression of osteoclast differentiation. In this culture model, we found that levels of full-length Luman increased transiently from day 1 to day 2 after treatment with RANKL and were then downregulated from day 3 (Fig. 1E, upper panel). However, the active fragments of the Luman N-terminus (hereafter referred to as Luman N-terminal fragments) were undetectable when BMMs were not treated with MG132. Following MG132 treatment, the Luman N-terminal fragments were successfully detected at days 1 and 2 in BMMs that had been treated with RANKL, indicating that the Luman N-terminus is degraded by the proteasome immediately after it has been cleaved from the full-length protein (Fig. 1F). Taken together, the results show that Luman is transiently induced in BMMs and cleaved in response to RANKL signaling during osteoclastogenesis.

**Fig. 2.**
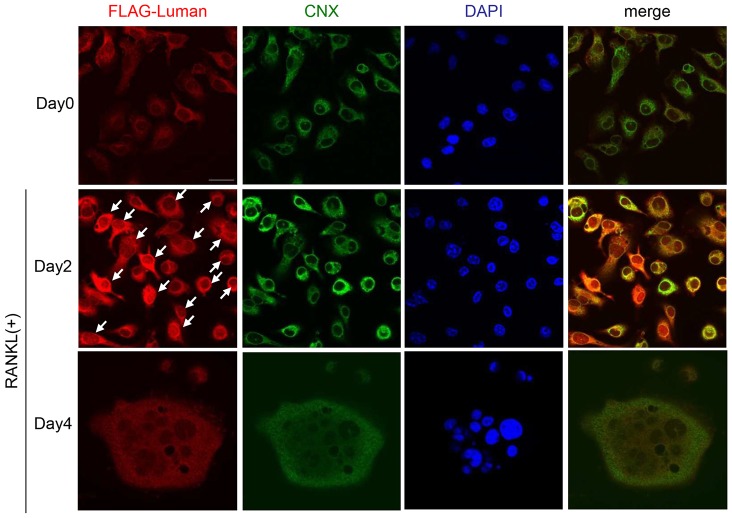
**Subcellular localization of Luman during osteoclastogenesis.** BMMs were infected with a retroviral vector expressing FLAG-tagged Luman. After viral infection, BMMs were cultured with M-CSF for 2 days. Thereafter, infected BMMs were incubated with M-CSF and RANKL for the indicated time periods. Cells were treated with proteasome inhibitor MG132 4 h before fixation, and then were fixed with cold methanol. Immunostaining was performed with antibodies against FLAG and calnexin (CNX), an ER marker. Nuclear counter-staining was conducted with DAPI. At day 2, signals for Luman in the nucleus as well as in the ER were detected (arrows). Scale bar: 20 µm.

Next, we analyzed the subcellular localization of Luman in osteoclasts. We used a retroviral expression vector to express FLAG-tagged full-length Luman (FLAG–Luman) in BMMs, and then treated the cells with RANKL for 2 or 4 days. Cells were treated with MG132 for 4 h before fixation for immunostaining. On day 0, prior to RANKL treatment, FLAG–Luman was detected in a reticular pattern in the cells (Fig. 2), completely overlapping with the staining of calnexin, an ER marker, indicating that Luman localizes to the ER in BMMs under steady-state conditions before RANKL treatment. On day 2 after RANKL treatment, FLAG–Luman was mainly detected in the nucleus (Fig. 2), whereas by day 4, FLAG–Luman expression was located only in the ER. Without MG132 treatment, signals of FLAG–Luman were undetectable at the nucleus throughout osteoclastogenesis (Fig. S1B). Considering these results, we conclude that Luman is cleaved in response to RANKL stimulation, whereby the N-terminal fragment translocates to the nucleus during the early stages of osteoclastogenesis and is then rapidly degraded.

### Luman is involved in cell–cell fusion of osteoclasts

**Fig. 3.**
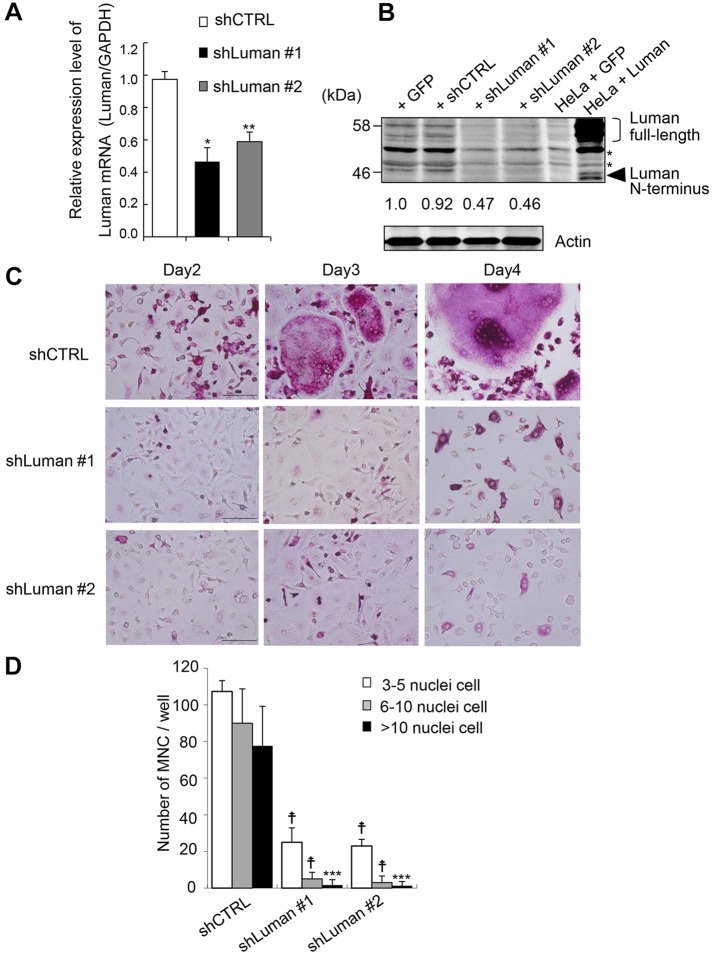
**Knockdown of Luman prevents osteoclast multinucleation.** (A) BMMs were infected with control shRNA retroviral vector (shCTRL) or shRNA retroviral vectors against (shLuman #1, shLuman #2). The expression levels of Luman were determined with real-time PCR analysis. Data are from four independent experiments. Values indicate mean±s.e.m. *P<0.05; **P<0.01. (B) BMMs were infected with control retroviral vector (GFP), shCTRL, shLuman #1 or shLuman #2). Each cell lysate was subjected to western blot analysis with an antibody against Luman. Cell lysate from Luman-transfected HeLa cells was used as a positive control. Asterisks indicate a non-specific band. Relative quantified data of bands representing full-length Luman were indicated below the Luman blot. The intensity of the band representing full-length Luman in the control sample was set as 1.0. (C) BMMs that had been infected with shCTRL or shLuman constructs were cultured with M-CSF and RANKL for the indicated periods. After incubation, cells were fixed and stained with TRAP-staining solution. Scale bars: 100 µm. (D) TRAP-positive multinucleated cells (MNC) with more than three nuclei were counted. Comparison of the number of TRAP-positive multinucleated cells between shCTRL- and shLuman-expressing cells revealed a significant decrease in the number of multinucleated cells in shLuman-infected cells. Data are from three independent experiments. Values indicate mean±s.d. ^†^*P*<0.001; ***P<0.005.

To investigate the function of Luman in osteoclastogenesis, we performed knockdown of Luman in osteoclasts. BMMs were infected with a retroviral vector expressing a small hairpin (sh)RNA against Luman or against the firefly luciferase gene as a control (shLuman and shCTRL, respectively). The mRNA and protein levels of Luman largely decreased following the introduction of shLuman (Fig. 3A,B). By day 3 of treatment with RANKL, numerous multinucleated cells were observed in shCTRL-infected cells (Fig. 3C; Fig. S1C, upper panels). By contrast, multinucleated cell formation was disturbed in cells in which Luman had been knocked down, and those cells remained as mononuclear cells until day 4 after stimulation with RANKL (Fig. 3C; Fig. S1C, lower panels). Although a large number of TRAP-positive mononuclear cells were observed in shLuman-expressing cells, TRAP-positive multinucleated cells were scarcely detected in those samples (Fig. 3C,D). From these results, pre-osteoclast formation proceeds normally, whereas multinucleation is impaired in Luman-knockdown cells, and Luman might positively regulate osteoclastogenesis.

### Luman induces the expression of DC-STAMP

**Fig. 4.**
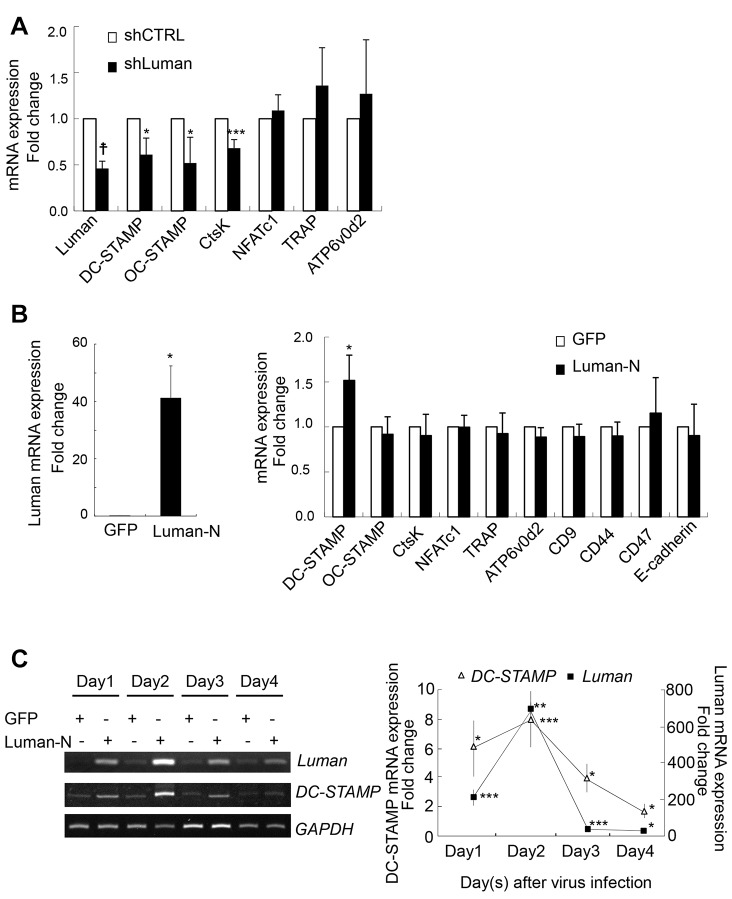
**Luman regulates *DC-STAMP* expression during osteoclastogenesis.** (A) BMMs that had been infected with shCTRL or shLuman #1 (shLuman) retroviral vectors were cultured with M-CSF and RANKL for 2 days. The expression levels of osteoclast genes were determined by using real-time PCR analysis. Data are from three independent experiments. Values indicate mean±s.e.m. *P<0.05; ***P<0.005; ^†^*P*<0.001. (B) BMMs were infected with GFP- or Luman-N-terminus-expressing retroviral vectors (GFP and Luman-N, respectively). After viral infection, BMMs were cultured with only M-CSF for 4 days. The expression levels of Luman and osteoclast genes were determined by real-time PCR analysis. Left, Luman mRNA expression levels in GFP or Luman-N infected cells. Right, mRNA expression levels of each gene in GFP- (□) or Luman-N-infected (▪) cells. Data are from three independent experiments. Values indicate mean±s.e.m. *P<0.05. (C) BMMs that had been infected with GFP- or Luman-N-terminus-expressing retroviral vectors were cultured with only M-CSF for the indicated periods. The expression levels of Luman and *DC-STAMP* mRNA were determined by using RT-PCR (left) and real-time PCR (right) analyses. Quantification chart indicates the fold induction relative to that of GFP-expressing samples. Data are from three independent experiments. Values indicate mean±s.e.m. *P<0.05; **P<0.01; ***P<0.005.

To address the molecular mechanism responsible for Luman-promoted osteoclastogenesis, we examined the expression of various genes that are involved in this differentiation process. BMMs that had been infected with retroviral shCTRL or shLuman vectors were treated with RANKL for 2 days and then analyzed for changes in the expression of various mRNAs using real-time PCR analysis. Intriguingly, knockdown of Luman resulted in a significant reduction in the expression of *CtsK*, *OC-STAMP* and *DC-STAMP* (Fig. 4A). Next, we tested the impact of Luman overexpression on the expression of osteoclast genes. We found an increase in the expression of *DC-STAMP* upon overexpression of the Luman N-terminus, but no change in *CtsK* or *OC-STAMP* expression (Fig. 4B). The expression pattern of *DC-STAMP* induction correlated well with the levels of exogenously expressed Luman N-terminus (Fig. 4C), suggesting that Luman directly promotes transcription of *DC-STAMP*, and that the decreased expression of *CtsK* and *OC-STAMP* is not due to direct effects of Luman knockdown.

**Fig. 5.**
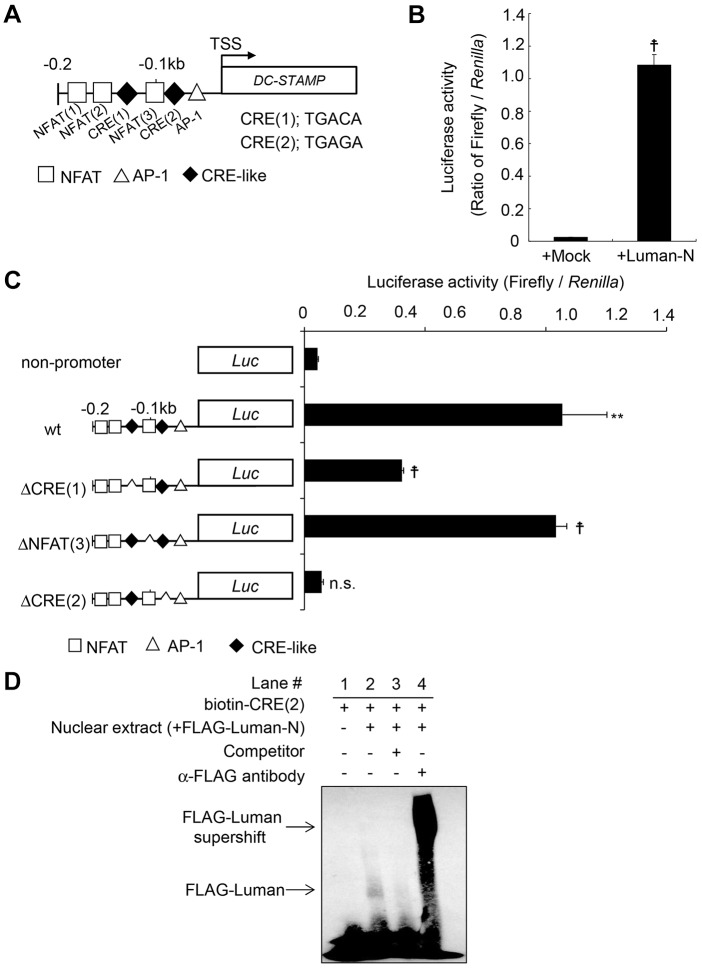
**Luman mediates the induction of *DC-STAMP* through CRE-like sequence in the *DC-STAMP* promoter.** (A) Schematic representation of the 0.2-kb promoter region of the murine *DC-STAMP* gene. AP-1 site (△), NFAT site (□) and CRE-like sequence site (◆) are indicated. TSS, transcription start site. Each CRE-like sequence is indicated. (B) The reporter plasmid of the 0.2-kb murine *DC-STAMP* promoter fused with the luciferase gene was co-transfected with the pcDNA empty vector (Mock) or Luman N-terminus pcDNA expression vector (Luman-N) into RAW264 cells. Luciferase activity was measured at 24 h after transfection. Data are from three independent experiments. Values indicate mean±s.e.m. ^†^*P*<0.001. (C) Luciferase reporter assay with a series of deletion mutants of the *DC-STAMP* promoter reporter plasmids. Reporter plasmids in which the NFAT-binding site or each CRE-like sequence had been deleted from the *DC-STAMP* promoter region were used. Reporter activities were measured in the same way as described in B. Data are from three independent experiments. Values indicate mean±s.e.m. **P<0.01; ^†^*P*<0.001; n.s., not significant. (D) Electrophoretic mobility shift assay. The biotin-labeled probes, including the CRE(2) site of the *DC-STAMP* promoter, were incubated with nuclear extract derived from FLAG–Luman-N expressing HeLa cells. Note that the binding of FLAG–Luman-N to the CRE(2) site was abolished by a competitor (lane 3), and a supershift after incubation with anti-FLAG antibodies (lane 4) was detected.

The 0.2-kb *DC-STAMP* promoter region includes one AP-1 site and three putative NFAT-binding sites to which c-Fos and NFATc1 bind, respectively (Yagi et al., 2007). Upon sequence analysis of the 0.2-kb *DC-STAMP* promoter 5′-upstream flanking region of the transcriptional start site of *DC-STAMP*, we found two putative cyclic AMP response element (CRE)-like sequences – 5′-TGACA-3′ [CRE(1)] and 5′-TGAGA-3′ [CRE(2)] (Fig. 5A). It is known that CRE sequences are potential sites through which OASIS-family members bind to and induce transcription of target genes (Asada et al., 2011). Therefore, it is possible that Luman induces the expression of *DC-STAMP* through the CRE-like sequence in the *DC-STAMP* promoter region by using the same mechanism as OASIS-family members. To examine whether Luman regulates *DC-STAMP* transcription directly, a promoter assay using a luciferase reporter plasmid driven by the *DC-STAMP* promoter region was performed. RAW264 cells were co-transfected with *DC-STAMP* promoter cloned into the pGL3 reporter plasmid and the expression plasmid encoding the the Luman N-terminus. The Luman N-terminus increased the promoter activities of *DC-STAMP* by approximately 50-fold, as compared with the control (Fig. 5B). An additional promoter assay was performed using a series of deletion-mutant reporter plasmids, in which the NFAT-binding site or each CRE-like sequence had been deleted. The reporter activities in cells that had been transfected with reporter plasmid lacking the NFAT-binding site in the *DC-STAMP* promoter region were almost the same as those of the wild-type reporter plasmid (Fig. 5C). Deletion of first CRE-like sequence in the promoter region still had an ability to elevate the *DC-STAMP* promoter activities (Fig. 5C). In contrast, deletion of the second CRE-like sequence [CRE(2), the one closest to transcription start site] in the promoter region diminished the increased of reporter activity induced by the Luman N-terminus (Fig. 5C). We also conducted electrophoretic mobility shift assays to investigate whether the Luman N-terminus binds to the second CRE-like sequence in the *DC-STAMP* promoter to regulate *DC-STAMP* transcription. The signal from a biotinylated CRE(2) probe was shifted upon incubation with the nuclear extract fraction from HeLa cells expressing exogenous FLAG-tagged Luman N-terminus (Fig. 5D, lane 2). This mobility shift was diminished by the addition of a competitor nucleic acid (Fig. 5D, lane 3). Additionally, super-shifting of the signal was observed following addition of antibodies against FLAG (Fig. 5D, lane 4). Collectively, these results indicate that the Luman N-terminus binds to the *DC-STAMP* promoter region. Thus, the second CRE-like sequence in the *DC-STAMP* promoter region is crucial for the regulation of *DC-STAMP* expression through the Luman N-terminus.

### The Luman–DC-STAMP signaling pathway plays an essential role in osteoclast multinucleation

**Fig. 6.**
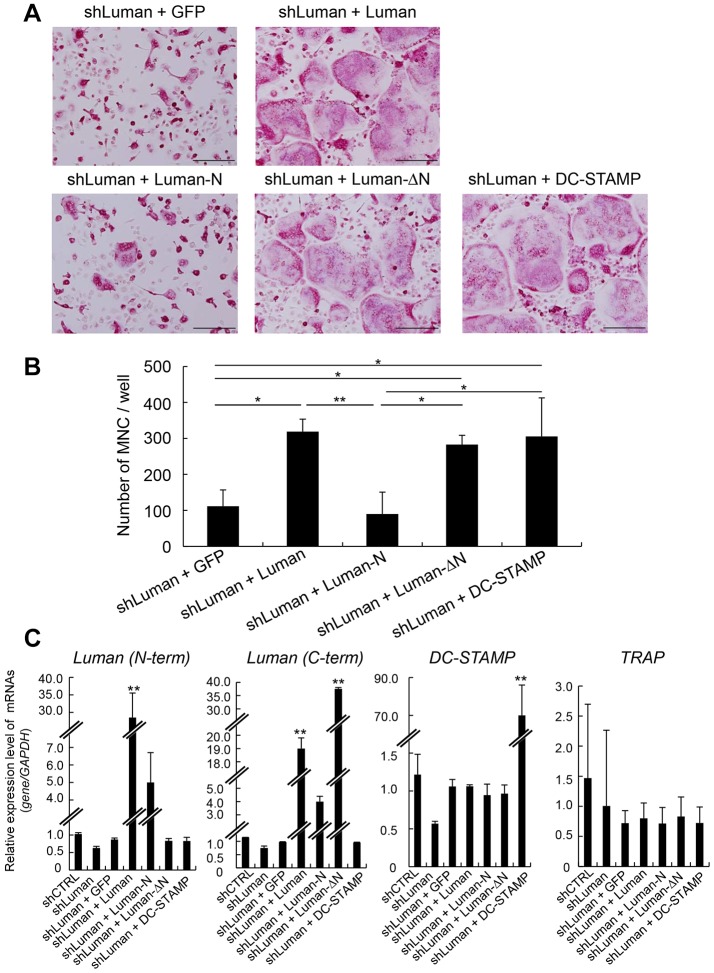
**Luman is involved in multinuclear osteoclast formation through the regulation of *DC-STAMP* expression.** (A) BMMs in which Luman had been knocked down were infected with retroviral vectors encoding GFP, various types of Luman or DC-STAMP. Thereafter, infected BMMs were incubated with M-CSF and RANKL for 3 days. After incubation, cells were fixed and stained with TRAP-staining solution. Scale bars: 200 µm. (B) TRAP-positive multinucleated cells (MNCs) with more than three nuclei from samples represented in A, were counted. Data are from three independent experiments. Values indicate mean±s.d. *P<0.05; **P<0.01 [one-way ANOVA with post-hoc Tukey honest significant difference (HSD) test]. (C) Real-time PCR analysis of Luman, *DC-STAMP* and *TRAP* mRNA. Luman N-terminal (N-term) primers detect the mRNA generated from Luman full-length and Luman N-terminus vectors. Luman C-terminal (C-term) primers detect the mRNA generated from Luman full-length and Luman-ΔN vectors. Note that the expression levels of *TRAP* mRNA were not altered even when Luman was introduced into shLuman-expressing BMMs. Data are from three or four independent experiments. Values indicate mean±s.d. **P<0.01 vs control (shCTRL) (one-way ANOVA with post-hoc Tukey HSD test).

As described above, DC-STAMP is known to be essential for the multinucleation of osteoclasts. Further, we have demonstrated that Luman affects the expression of *DC-STAMP* at the transcriptional level. Therefore, we next investigated whether the Luman–DC-STAMP signaling pathway has an impact on multinucleation during osteoclast differentiation. Introduction of full-length Luman into shLuman-expressing BMMs rescued the perturbed multinucleation of osteoclasts (Fig. 6A, upper-right panel). We also found that introduction of DC-STAMP into shLuman-expressing BMMs induced the multinucleation of osteoclasts. Addition of the Luman N-terminus did not rescue these multinucleation defects, whereas introduction of N-terminus-deleted Luman mutant – which lacks the transcription activation domain of Luman (Luman-ΔN) – did rescue the defects (Fig. 6A, bottom panels). The number of multinucleated cells was also significantly increased following introduction of full-length Luman, DC-STAMP and Luman-ΔN, but not the Luman N-terminus (Fig. 6B). The expression level of *DC-STAMP* was recovered to that of control by restoring Luman (Fig. 6C). However, the expression levels of *TRAP* were similar in cells expressing each of the constructs to those in the control sample (Fig. 6C). The above findings suggest that Luman acts at the final stage of osteoclastogenesis by facilitating osteoclast terminal differentiation. Although the N-terminus of Luman plays a key role in the induction of DC-STAMP, the transcriptional activities of Luman cannot account for the induction of osteoclast multinucleation. Rather, either the full-length protein or a region other than the N-terminus appears to be necessary for multinucleation.

### Luman interacts with DC-STAMP, and they colocalize at the ER and Golgi

**Fig. 7.**
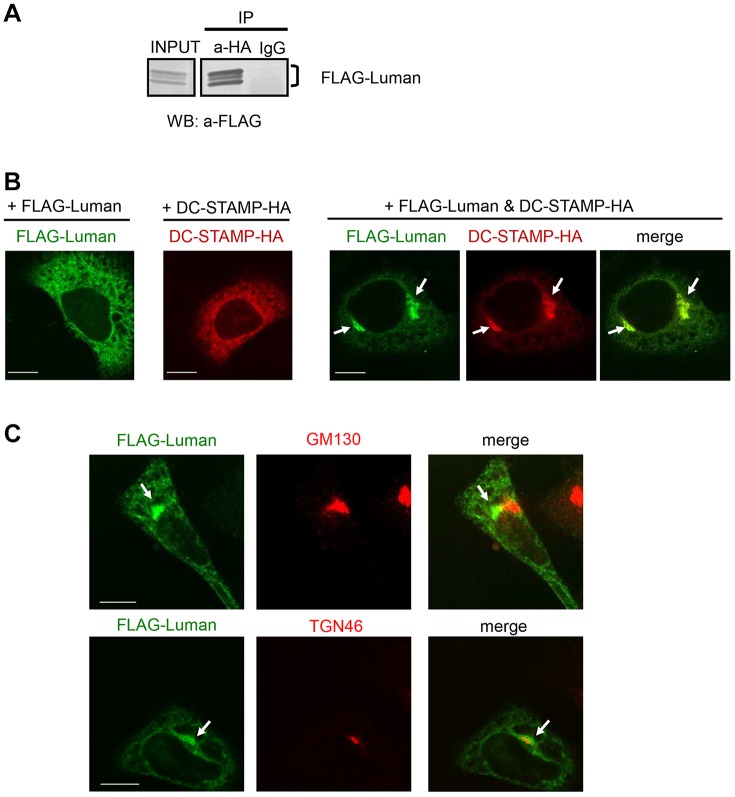
**Luman interacts with DC-STAMP and is localized to the Golgi.** (A) Co-immunoprecipitation analyses of HeLa cells expressing Luman and DC-STAMP. Cells were co-transfected with expression plasmids for Luman tagged with FLAG at the N-terminus (FLAG–Luman) and DC-STAMP tagged with HA at the C-terminus (DC-STAMP–HA). Cell lysates were immunoprecipitated with antibodies against HA (a-HA), and the immunoprecipitated (IP) samples were subjected to western blotting (WB) with an antibody against FLAG. (B) Subcellular localization of Luman and DC-STAMP that had been expressed in HeLa cells. FLAG–Luman and/or DC-STAMP–HA expression plasmids were transfected into HeLa cells. The transfected cells were immunostained with antibodies against FLAG or HA. Note that co-expression of Luman and DC-STAMP caused the emergence of perinuclear accumulation. Arrows indicate accumulated signals of Luman and DC-STAMP. Scale bars: 10 µm. (C) Double-staining for FLAG and Golgi markers on the cells expressing FLAG–Luman and DC-STAMP–HA. HeLa cells were co-transfected with FLAG–Luman and DC-STAMP–HA expression plasmids. The transfected cells were immunostained with antibodies against FLAG and GM130 (cis-Golgi marker) or against FLAG and TGN46 (trans-Golgi marker). Note that perinuclear accumulation of FLAG–Luman and DC-STAMP–HA overlaps with that of the trans-Golgi marker. Arrows indicate accumulated signals for Luman and DC-STAMP. Scale bars: 10 µm.

HeLa cells were transfected with FLAG–Luman and hemagglutinin (HA)-tagged DC-STAMP (DC-STAMP–HA) expression plasmids to further analyze the functions of both molecules. Co-immunoprecipitation experiments for DC-STAMP–HA revealed that Luman bound to DC-STAMP in HeLa cells (Fig. 7A). This result is consistent with previously reported data (Eleveld-Trancikova et al., 2010), although the physiological functions of their interaction have yet to be clarified. Therefore, to assess the impact of the interaction between Luman and DC-STAMP on their functions, we analyzed their subcellular localizations. When FLAG–Luman or DC-STAMP–HA was introduced alone, each was expressed in a diffuse manner with an ER-localized pattern (Fig. 7B; Fig. S2A,C,D). Interestingly, after co-expression of Luman and DC-STAMP, the two molecules accumulated at the perinuclear region (Fig. 7B; Fig. S2A, arrows). Moreover, after treatment with MG132, the Luman signal was also detected at the nucleus in the cells expressing both Luman and DC-STAMP, suggesting that Luman might undergo RIP (Fig. S2A, arrowheads). To identify the region where Luman and DC-STAMP accumulate, cells expressing both molecules were immunostained for intracellular markers. As a result, the signals of Luman and DC-STAMP overlapped with those of a trans-Golgi network protein, TGN46 (Fig. 7C; Fig. S2B, arrows). Thus, when these two proteins interact with each other, the intracellular localization of both Luman and DC-STAMP can be changed from the ER to the Golgi.

### Luman regulates DC-STAMP localization and stability

**Fig. 8.**
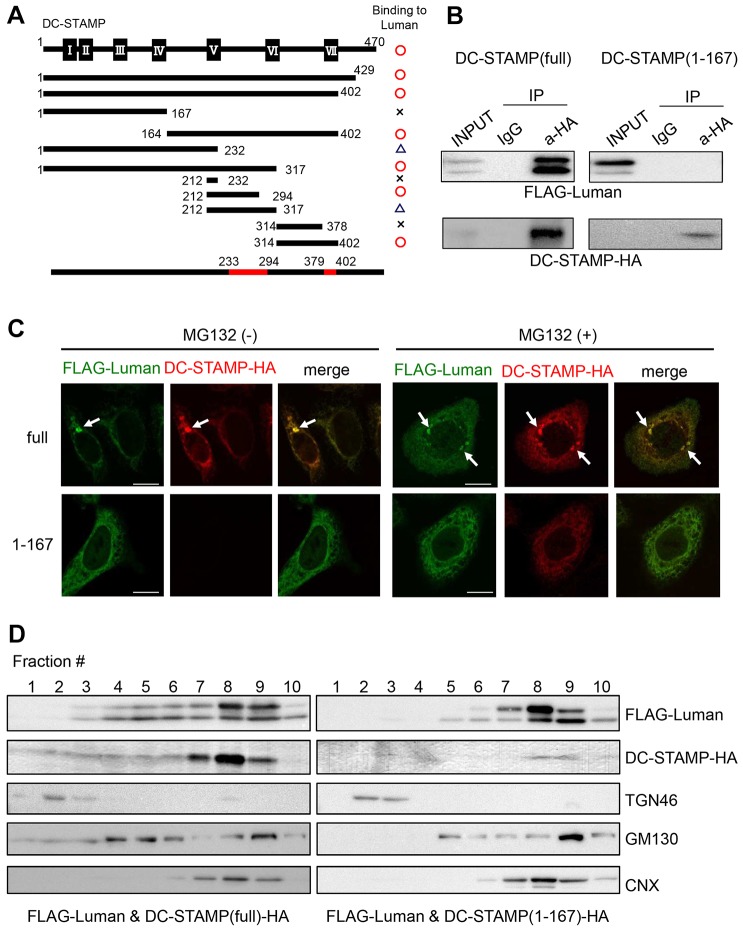
**Luman defines the localization and stabilization of DC-STAMP by interacting with it.** (A) Schematic representation of the domain structure of murine DC-STAMP and mapping of the binding region for Luman. Co-immunoprecipitation experiments with various truncated DC-STAMP mutants revealed the binding region in DC-STAMP for Luman. Solid squares with Roman numerals indicate the transmembrane domains of DC-STAMP. Putative binding regions are highlighted in red. The degrees of association between Luman and the DC-STAMP constructs shown are indicated as follows: circle, strong; triangle, weak; cross, none. Numbers represent amino acid residues. (B) Co-immunoprecipitation followed by western blot analysis. HeLa cells were co-transfected with expression plasmids for FLAG–Luman and full-length DC-STAMP [DC-STAMP(full)–HA] or HA-tagged truncated DC-STAMP [DC-STAMP(1-167)–HA]. Cell lysates were immunoprecipitated (IP) with antibodies against HA (a-HA), and the immunoprecipitated proteins were subjected to western blotting with antibodies against FLAG or HA. Note that Luman only interacts with the full-length DC-STAMP. (C) Immunostaining for FLAG–Luman and DC-STAMP–HA. HeLa cells were co-transfected with FLAG–Luman and DC-STAMP(full)–HA or DC-STAMP(1-167)–HA expression plasmids, and immunostained with antibodies against FLAG (green) and HA (red). Upon co-expression of FLAG–Luman and DC-STAMP(full)–HA, both proteins accumulated at the perinuclear region. DC-STAMP(1-167)–HA was scarcely detected in the absence of treatment with MG132. Arrows indicate accumulated signals for Luman and DC-STAMP. Scale bars: 10 µm. (D) Subcellular fractionation analyses of Luman and DC-STAMP. HeLa cells were co-transfected with expression plasmids for FLAG–Luman and DC-STAMP(full)–HA or DC-STAMP(1-167)–HA. The microsome membranes from cell lysates were ultracentrifuged and fractionated in an iodixanol gradient. Each fraction was subjected to western blotting. TGN46, GM130 and calnexin (CNX) were examined as specific intracellular markers for the trans-Golgi, cis-Golgi and ER, respectively. TGN46 is mainly distributed in fractions 2 and 3; GM130 in fractions 4–6; CNX in fractions 7–9.

To determine the binding region for Luman in DC-STAMP in detail, a series of deletion constructs of DC-STAMP tagged with HA at the C-terminus were generated. For protein interaction experiments, we used HeLa cells that lacked DC-STAMP expression in order to exclude the influence of endogenous DC-STAMP. Co-immunoprecipitation analyses with antibodies against HA revealed that the putative binding regions for Luman in DC-STAMP were amino acids 233–294 and 379–402 (Fig. 8A). Furthermore, the region of Luman that is responsible for binding to DC-STAMP was determined. Several truncated Luman constructs that had been tagged with FLAG at the N-terminus were produced and analyzed in a series of co-immunoprecipitation studies. These studies identified the transmembrane region of Luman as being necessary for the interaction with DC-STAMP (Fig. S3A,B). Next, we investigated whether truncated DC-STAMP, which shows diminished interaction with Luman, affects the intracellular localization of Luman. To examine this, we used an expression plasmid encoding DC-STAMP(1-167)–HA. The expression level of DC-STAMP(1-167)–HA was substantially weak, suggesting that this mutant DC-STAMP might be unstable. However, after immunoprecipitation with antibodies against HA, a signal for DC-STAMP(1-167)–HA was detected (Fig. 8B, lower-right panel). Co-immunoprecipitation analyses showed that FLAG–Luman interacted with full-length DC-STAMP–HA, as shown earlier, whereas FLAG–Luman was unable to bind to DC-STAMP(1-167)–HA (Fig. 8B, upper-left and upper-right panels). Upon immunostaining, signals for DC-STAMP(1-167)–HA were scarcely detected in the cells that had been transfected with both FLAG–Luman and DC-STAMP(1-167)–HA in the absence of treatment with MG132 (Fig. 8C). However, when the transfected cells were treated with MG132, signals for DC-STAMP(1-167)–HA were clearly detected (Fig. 8C). These findings suggest that this truncated mutant DC-STAMP, lacking the interaction with Luman, was readily degraded by the proteasome. We further examined the subcellular localizations of FLAG–Luman and DC-STAMP(1-167)–HA using HeLa cells that expressed both molecules. FLAG–Luman was distributed in a diffuse manner throughout the cell with or without MG132 treatment, indicating that FLAG–Luman was localized to the ER in the absence of an interaction with DC-STAMP (Fig. 8C, lower-left panel). DC-STAMP(1-167)–HA was only detected after treatment with MG132 (Fig. 8C). The distribution of DC-STAMP(1-167)–HA showed an ER-like pattern, similar to that of FLAG–Luman (Fig. 8C). In contrast, immunostaining of HeLa cells that expressed both FLAG–Luman and full-length DC-STAMP–HA revealed that the staining of the two proteins overlapped, and both were observed to accumulate at the perinuclear region, both with and without MG132 treatment (Fig. 8C, upper panels). To confirm the intracellular distributions of Luman and DC-STAMP, iodixanol-gradient fractionation was performed. Western blot analyses of the iodixanol-gradient-fractionated samples showed that FLAG–Luman that had been co-expressed with full-length DC-STAMP–HA in cells existed in the trans-Golgi, cis-Golgi and ER fractions (Fig. 8D; Fig. S3C, left panels). Full-length DC-STAMP–HA was also distributed in the trans-Golgi, cis-Golgi and ER fractions (Fig. 8D; Fig. S3C, left panels). In contrast, when the cells co-expressed FLAG–Luman and DC-STAMP(1-167)–HA, the expression level of the mutant DC-STAMP became impaired, as previously mentioned (Fig. 8D; Fig. S3C, right panels). Although the expression of the mutant DC-STAMP was very weak, it was detectable and its distribution in cells was restricted to the ER (Fig. 8D; Fig. S3C, right panels). When Luman could not interact with DC-STAMP, it existed mainly in the ER and partly in the cis-Golgi (Fig. 8D; Fig. S3C, right panels). These results were consistent with the immunostaining data. Taken together, the results indicate that Luman and DC-STAMP interact with each other and might regulate their localization in a coordinated manner. More importantly, the stability of DC-STAMP might be defined by its interaction with Luman. In osteoclastogenesis, the localization of DC-STAMP is considered to be important for the cell–cell fusion step. Therefore, Luman might control the multinucleation step of osteoclast formation by regulating the localization of DC-STAMP (Fig. S4).

## DISCUSSION

In this report, we have shown that Luman is induced during osteoclastogenesis in response to RANKL signaling and that a reduction in the expression of Luman through shRNA knockdown perturbs the formation of multinucleated osteoclasts. However, staining of TRAP showed that there were numerous TRAP-positive mononuclear cells remaining in the culture, indicating that pre-osteoclast differentiation from monocytes and macrophages proceeded as normal. From these results, we concluded that Luman plays a role in regulating the cell–cell fusion step of osteoclastogenesis. DC-STAMP and ATP6v0d2 are essential molecules for cell–cell fusion of osteoclasts. Analyses of BMMs derived from DC-STAMP-deficient or ATP6v0d2-deficient mice has revealed inhibition of osteoclast multinucleation (Yagi et al., 2005; Lee et al., 2006). Recently, another molecule, OC-STAMP, has been identified as an essential factor for cell–cell fusion of osteoclasts and foreign body giant cells (FBGCs) (Miyamoto et al., 2012b). Multinucleation of osteoclasts is disturbed in OC-STAMP-deficient BMMs as well as in DC-STAMP-deficient cells (Miyamoto et al., 2012b). Our knockdown and overexpression analyses in BMMs revealed that Luman directly controls the expression of DC-STAMP, but not that of OC-STAMP or ATP6v0d2. The introduction of Luman into Luman-knockdown BMMs recovered the expression of DC-STAMP and multinucleation of osteoclasts, suggesting that Luman directly regulates the cell fusion of osteoclasts through the induction of DC-STAMP. However, expression of the Luman N-terminus failed to recover the multinucleation defects that were induced by Luman shRNA knockdown. Therefore, the transcriptional activity of Luman is insufficient for the induction of osteoclast multinucleation. This suggests that Luman has an additional function at the final stage of osteoclastogenesis.

We also demonstrated that Luman is cleaved at the membrane region and becomes activated during the early stages of osteoclast differentiation. The other OASIS-family members OASIS and BBF2H7 are activated during osteoblast and chondrocyte differentiation, respectively (Murakami et al., 2009; Saito et al., 2009). Activation of these molecules is dependent on physiological ER stress caused by the production of abundant proteins during cellular maturation. However, we could not detect any cleavage of Luman in cells that had been exposed to ER stress, but instead identified its cleavage and activation in response to RANKL signaling. These findings suggest that the cleavage and activation of Luman during osteoclastogenesis are not caused by ER stress, and that the mechanism of activation of Luman is different from that of OASIS and BBF2H7. During RIP, Luman is processed by S1P that is resident in the Golgi. Therefore, Luman has to be delivered to the Golgi in order to become activated after cells have received signals to differentiate into osteoclasts. When Luman was overexpressed alone in HeLa cells, it became localized at the ER. However, co-expression of Luman and DC-STAMP led to the interaction of both proteins and their transportation from the ER to the Golgi. Once Luman has moved to the Golgi, S1P in the Golgi body is able to access Luman and cleave it. Moreover, overexpression of both Luman and DC-STAMP in HeLa cells enabled signals from Luman to be detected in the nucleus after treatment with MG132. This suggests that DC-STAMP facilitates cleavage of Luman through transportation of Luman to the Golgi. Thus, it is possible that DC-STAMP functions as an escort protein and regulates the sorting of Luman to the Golgi body through a direct interaction with Luman. This hypothesis is further supported by the findings that Luman was not delivered to the Golgi after co-expression with mutant DC-STAMP that lacked the ability to bind to Luman. It has been reported that Luman and DC-STAMP interact with OS-9, which is involved in ER-associated degradation (ERAD) (Eleveld-Trancikova et al., 2010). Association with or dissociation from OS-9 might be a trigger for the protein delivery or degradation of Luman and/or DC-STAMP. The evidence that the mutant DC-STAMP lacking the interaction with Luman was degraded immediately by the ubiquitin-proteasome system supports this notion. Thus, a complex of Luman–DC-STAMP–OS-9 is likely to regulate the localization and stability of each protein. Mammalian and insect cells possess a similar transport regulation system, in which the SREBP–SCAP–Insig complex regulates cholesterol synthesis. SREBP is a membrane-resident transcription factor that controls the transcription of enzymes involved in the production of cholesterol (Rawson, 2003). Under normal conditions, SREBP, SCAP and Insig form a tripartite complex and remain in the ER membrane. However, once the cholesterol amount in cells becomes reduced, one of the members in the complex, Insig, dissociates from the complex, and the resulting binary complex of SREBP and SCAP is transported to the Golgi from the ER. Subsequently, SREBP is processed by S1P and site-2 protease during RIP, and the cleaved fragment of SREBP functions as a transcription factor. The Luman–DC-STAMP–OS-9 complex seems to function in similar manner to that of the SREBP–SCAP–Insig complex, and might control the transport of Luman and DC-STAMP to their proper intracellular positions in response to specific signaling, such as RANKL stimuli. This trafficking regulation would be a key point for the activation of Luman. Furthermore, DC-STAMP is needed for the sorting of Luman to the Golgi, whereas conversely, Luman is required for the delivery of DC-STAMP from the ER to the Golgi and subsequently to the plasma membrane. Based on our findings, a model in which the two molecules interact with each other to regulate each other's functions can be proposed (Fig. S4).

Recent reports have suggested that regulation of DC-STAMP localization is important for normal cell–cell fusion of osteoclasts and FBGCs (Hobolt-Pedersen et al., 2014; Islam et al., 2014). DC-STAMP is localized at the surface area in pre-osteoclasts and small osteoclasts to initiate cell–cell fusion, whereas it is present in internal areas in mature large multinucleated osteoclasts (Hobolt-Pedersen et al., 2014). It has been reported that Pin1, a peptidylprolyl isomerase, regulates the localization of DC-STAMP (Islam et al., 2014). Inhibition of Pin1 causes retention of DC-STAMP in the cytosolic region, and Pin1-deficient osteoclasts become larger than wild-type osteoclasts (Islam et al., 2014). These observations suggest that DC-STAMP functions in the early stage of cell–cell fusion and that the localization of DC-STAMP is important for normal osteoclast cell–cell fusion. Therefore, from our present data, there is a possibility that the suppression of multinucleated osteoclast formation through Luman knockdown is caused by a failure of DC-STAMP transport.

In conclusion, we have newly identified Luman as a key factor that is involved in osteoclastogenesis. Luman functions as a regulator of multinucleated cell formation through the control of DC-STAMP expression, stability and localization. This provides new evidence that signaling from the ER is involved in osteoclast formation. Although further careful investigations are required, Luman might become a therapeutic target for osteoclast-related diseases, including osteoporosis, osteopetrosis and rheumatoid arthritis.

## MATERIALS AND METHODS

### Reagents and antibodies

M-CSF and soluble RANKL were purchased from Kyowa Hakko-Kirin and Oriental Yeast, respectively. MG132 was purchased from Wako. TRAP-staining kit was obtained from Sigma-Aldrich. Anti-Luman antibody (sc-25074, Santa Cruz Biotechnology), anti-calnexin antibody (MAB3126, Chemicon International), anti-FLAG antibody (F3165, Sigma), anti-HA antibody (2367S, 3724S, Cell Signaling Technology), anti-GM130 antibody (610822, BD Transduction Laboratories) and anti-TGN46 antibody (ab16509, Abcam, Cambridge) were used for western blotting or immunofluorescence staining.

### Animals

Male C57BL/6 or ICR mice, aged 4–6 weeks, were purchased from Charles River Laboratories, Japan. The experimental procedures and housing conditions for animals were approved by the Committee of Animal Experimentation, Hiroshima University.

### Osteoclast formation and TRAP staining

BMMs were isolated from tibiae of mice and cultured in α minimum essential medium (α-MEM) supplemented with 10% fetal bovine serum (FBS) in the presence of 50 ng/ml M-CSF for 17 h. Floating BMMs were collected, and 7.5×10^4^ cells were seeded in each well of a 96-well culture plate and cultured in the presence of M-CSF for 2 days. Thereafter, adhered BMMs were cultured with 50 ng/ml M-CSF and 100 ng/ml RANKL for 4 days. The culture medium was changed on day 2. For the evaluation of osteoclast formation, TRAP staining was performed according to the manufacturer's protocol.

### Cell culture and transfection

Murine ATDC5 chondrocyte cells, murine MC3T3-E1 osteoblast-like cells and murine RAW264 macrophage cells were cultured in α-MEM supplemented with 10% FBS. RAW264 cells were a kind gift from Dr Yuki Imai (Ehime University, Matsuyama, Japan). HeLa cells and Plat-E cells were maintained in Dulbecco's modified Eagle's medium (DMEM) supplemented with 10% FBS. Plat-E cells were graciously provided by Dr Toshio Kitamura (The University of Tokyo, Tokyo, Japan). HeLa cells or RAW264 cells were transfected with Lipofectamine2000 (Invitrogen), according to the manufacturer's protocol.

### RNA extraction, reverse transcription PCR and real-time PCR

Total RNA was extracted from cell lines, osteoclasts or BMMs with ISOGEN (Nippongene), according to the manufacturer's protocol. cDNA was synthesized from 1 μg of total RNA as a template using M-MLV reverse transcriptase (Invitrogen). Reverse transcription (RT)-PCR analysis was performed as described previously (Kudo et al., 2008). Real-time PCR analysis was conducted using a LightCycler 480 (Roche) and KAPA SYBR FAST qPCR kit (KAPA Biosystems). *GAPDH* served as an internal control. Primers for RT-PCR and real-time PCR analyses are indicated in Table S1.

### Plasmids, retrovirus preparation and infection

Murine Luman cDNA was cloned from BMM mRNA by using PCR. Primer sets for cloning are indicated in Table S1. Obtained cDNAs were cloned into the pMX retroviral vector or the pcDNA3.1(+) expression vector using *Eco*RI. The pMX vector was kindly provided by Dr Toshio Kitamura. The DC-STAMP expression vector was prepared as described before (Sawatani et al., 2008). A series of truncated mutant DC-STAMP or Luman expression plasmids were constructed by using PCR and cloned into pcDNA3.1(+) vectors. For the knockdown of Luman, we generated a viral vector by cloning Luman shRNA into the retroviral pSINsi-U6 vector (TaKaRa Bio), using the following primers, where the target sequences are indicated in capital letters: mouse Luman (#1), 5′-AGAAGAAGCTCTTGGAGAActgtgaagccacagatgggTTCTCCAAGAGCTTCTTCTtttttt-3′; mouse Luman (#2), 5′-ACAGGAGATGTCTAGGCTGATctgtgaagccacagatgggATCAGCCTAGACATCTCCTGTtttttt-3′; luciferase, 5′-GTTGGCACCAGCAGCGCACctgtgaagccacagatgggGTGCGCTGCTGGTGCCAACtttttt-3′. The reporter plasmid, driven by the mouse *DC-STAMP* promoter region, was prepared as described previously (Yagi et al., 2007). Deletion mutants of the DC-STAMP promoter region were produced by using PCR and cloned into pGL3-basic reporter plasmids. The pMX retroviral vector harboring the Luman, *DC-STAMP* or *GFP* cDNA was introduced into Plat-E retrovirus packaging cells (Morita et al., 2000) using X-tremeGENE9 (Roche). The viral supernatants were collected 48 h after transfection and passed through a 0.45-μm pore size syringe filter. BMMs were infected with viral supernatant mixed with M-CSF (25 ng/ml) and polybrene (4 μg/ml) for 24 h, and then infected BMMs were cultured with M-CSF (50 ng/ml) and RANKL (100 ng/ml) for 4 days.

### Protein analysis

Cultured cells, BMMs or osteoclasts were washed with ice-cold PBS and then lysed with SDS lysis buffer [33 mM Tris-Acetate (pH 8.5), 1.6% Triton-X 100, 0.3% SDS, 5 mM EDTA, 2.7 mM methionine and protein inhibitor cocktail (MLB)]. Protein concentration was measured using the bicinchoninic acid (BCA) assay (Pierce). Equal amounts of cell lysate were subjected to SDS-PAGE and then transferred to polyvinylidene difluoride (PVDF) membranes (Bio-Rad Laboratories) for western blotting. Membranes were blocked with 5% nonfat skim milk, followed by incubation with primary antibody overnight at 4°C. Horseradish-peroxidase-conjugated secondary antibodies (Jackson ImmunoResearch laboratories) were used to probe for specific primary antibodies, and the signal was developed with ECL solution (Bio-Rad) and analyzed by using VersaDoc (Bio-Rad). Primary antibodies against Luman and actin were obtained from Santa Cruz Biotechnology and Millipore, respectively.

### Immunofluorescence staining

BMMs were infected with a retroviral vector expressing FLAG-tagged Luman, and osteoclasts were generated as described above. Cells were fixed in cold 100% methanol for 10 min, washed three times with phosphate-buffered saline (PBS), and then permeabilized in 0.5% Triton-X 100 diluted in PBS. Cells were incubated overnight with anti-FLAG M2 (SIGMA) and anti-calnexin (Enzo Life Sciences) antibodies diluted 1:200 at 4°C, then with Alexa-Fluor-546-conjugated anti-mouse IgG antibodies (Invitrogen) and FITC-conjugated anti-rabbit IgG antibodies (Molecular Probes) diluted 1:500 for 2 h at room temperature. Staining was then visualized under a confocal microscope (Olympus FV1000D). Nuclei were counterstained with DAPI (Molecular Probes). HeLa cells were transfected with FLAG–Luman and/or DC-STAMP–HA expression plasmids. Immunostaining was performed as described above. For primary antibodies, anti-FLAG M2, anti-HA, anti-GM130 and anti-TGN46 antibodies were used.

### Luciferase assay

RAW264 cells were plated at 1×10^5^ cells per well in a 24-well plate 1 day before transfection. Cells were then transfected using FuGene HD (Promega) with the *DC-STAMP*-promoter–reporter plasmid (0.2 μg) carrying the firefly luciferase gene, a reference plasmid pRL-SV40 (0.02 μg) carrying the *Renilla* luciferase gene under the control of the SV40 enhancer and promoter (Promega), and an effector protein expression plasmid (0.2 μg). After 24 h, luciferase activity was measured using Dual-Luciferase Reporter Assay System (Promega) and a luminometer (Promega), according to the manufacturer's protocol. Relative activity was defined as the ratio of firefly luciferase activity to that of *Renilla* luciferase.

### Electrophoretic mobility shift assay

The following oligonucleotides were used as probes: DC-STAMP pro CRE2-fwd: 5′-GTGGAGGAAA**TGAGA**AGATTGATTCA-3′ and DC-STAMP pro CRE2-rev: 5′-TGAATCAATCT**TCTCA**TTTCCTCCAC-3′ (the CRE-like sequences are shown in bold). Double-stranded synthetic oligonucleotides were labeled with biotin. The FLAG-tagged Luman N-terminus expression plasmid was transfected into HeLa cells, and nuclear extracts were isolated. The electrophoretic mobility shift assay was performed using Chemiluminescent Nucleic Acid Detection Module (Thermo Scientific), according to the manufacturer's protocol. For supershift experiments, samples were treated with an anti-FLAG antibody (Rockland) at 4°C for 1 h before incubation with a biotin-labeled probe.

### Co-immunoprecipitation assay

HeLa cells were co-transfected with Luman tagged with FLAG at the N-terminus (FLAG–Luman) and DC-STAMP tagged with HA at the C-terminus (DC-STAMP–HA) expression plasmids. Cells were harvested at 24 h after transfection, and then lysed with SDS lysis buffer. Cell lysates were immunoprecipitated with anti-HA rabbit monoclonal antibodies together with rProteinG Agarose Beads (Invitrogen). Beads were washed with 1× TBS buffer (25 mM Tris-HCl, pH 7.5, 137 mM NaCl, 2.7 mM KCl supplemented proteinase inhibitors) five times, and then immunoprecipitated samples were subjected to western blotting with anti-FLAG mouse monoclonal antibodies or anti-HA mouse monoclonal antibodies.

### Fractionation of microsomal membrane

HeLa cells were co-transfected with FLAG–Luman and DC-STAMP–HA expression plasmids. Cells were harvested at 24 h after transfection and then homogenized with 26 G needle in HEPES buffer (5 mM HEPES, pH 7.4, 1 mM EDTA and a protease inhibitor mixture from Wako). Cell homogenates were centrifuged at 1000 ***g*** for 10 min, and the post nuclear supernatant was then centrifuged again at 3000 ***g*** for 10 min. The supernatant was collected as a microsomal fraction. Discontinuous iodixanol gradients in HEPES buffer were prepared by layering 0.5 ml each of 2.5, 5, 7.5, 10, 12.5, 15, 20, 25 and 30% of OptiPrep (Sigma) in a centrifuge tube. The microsomal fraction was applied to the top of iodixanol gradient. Following ultracentrifugation (90,000 ***g***, 16 h, 4°C), 0.5-ml fractions were collected and analyzed by western blotting. Specific markers for the ER (calnexin), cis-Golgi (GM130) and trans-Golgi (TGN46) were used.

### Statistical analysis

Data are indicated as mean±s.d. or s.e.m. from at least three independent experiments for each experimental condition. Student's *t*-test or one-way analysis of variance (ANOVA) was performed to calculate *P* values. *P*<0.05 was defined as significant.
